# Circadian rhythm dysregulation and brain functional connectivity alterations in stroke patients: an exploratory study of peripheral and central clock interactions

**DOI:** 10.1186/s12883-026-05205-9

**Published:** 2026-08-14

**Authors:** Xiaoli Wu, Fan Bai, Lu Zhang, Lixu Liu, Yudong Chen, Hanzhi Li, Tong Zhang, Yanning Cai

**Affiliations:** 1 Department of Neurorehabilitation, Rehabilitation Research Centre, Rehabilitation Medicine of Capital Medical University, No. 10, Jiaomen North Road street, Fengtai District, Beijing, 100068 China; 2 Rehabilitation Medicine Research Department, Beijing Key Laboratory of Neural Injury and Rehabilitation, China Rehabilitation Science Institute, Beijing, China; 3 Neurobiology Lab, Key Laboratory of Neurodegenerative Diseases, Ministry of Education, Xuanwu Hospital of Capital Medical University, Beijing, 100053 PR China

**Keywords:** Stroke, Circadian rhythm, Resting functional magnetic resonance imaging, Functional connectivity, Bmal1

## Abstract

**Background:**

The human circadian rhythm system is governed by a transcriptional-translational feedback loop of clock genes, including *Bmal1*, which exhibits a near-24-hour oscillatory period. Emerging evidence highlights the critical contribution of circadian genes to stroke pathogenesis and outcomes, yet mechanistic insights into their regulatory roles remain limited. To bridge this knowledge gap, we aimed to determine whether the serum of stroke patients could affect changes in the molecular clock and whether such changes in the molecular clock were potentially linked to abnormal brain functional connectivity in stroke patients.

**Methods:**

This prospective case-control study included 17 patients with stroke and 17 age-matched healthy participants. All participants were required to complete a standardized comprehensive questionnaire. Transfected human osteosarcoma cells (U2OS) stably expressing *Bmal1-dluc* reporters were treated with serum from both groups to monitor changes in the daily oscillation period of Bmal1. We also compared the changes in resting-state hypothalamic intrinsic functional connectivity(FC) between the two groups. Further, we explored the correlation between the differences in FC between the two groups and the daily oscillation period of *Bmal1*.

**Results:**

Compared to the healthy control group, stroke patients exhibited a significantly prolonged peripheral *Bmal1* circadian oscillation period (24.32 ± 0.41 h vs. 23.81 ± 0.48 h, t = 3.352, *p* = 0.002). In FC analysis, the stroke group showed enhanced connectivity from the hypothalamus to the right occipital lobe and the right insular region. Notably, the degree of this connectivity enhancement demonstrated a significant positive correlation with the length of *Bmal1* oscillation period (*r* = 0.369, *p* = 0.032; *r* = 0.393, *p* = 0.022).

**Conclusion:**

Our findings suggest that serum from stroke patients lengthens the circadian period in U2OS cells and may be associated with alterations in brain functional connectivity. However, the small sample size limits statistical power, and these exploratory results require replication in larger independent cohorts.

**Trial registration:**

ChiCTR2000031671(April 4, 2020).

## Background

Recent studies have revealed that circadian rhythm, as an endogenous timing system, exerts multidimensional regulatory effects throughout the entire course of stroke occurrence, progression, and rehabilitation [1, 2]. It directly contributes to stroke pathophysiology by modulating vascular endothelial function, lipid/glucose metabolism, coagulation cascades, vascular tone, microglial activation, and neuroinflammatory responses [3–6]. A bidirectional interaction exists between circadian rhythm and ischemic stroke: stroke reciprocally disrupts sleep patterns, endogenous rhythms, and circadian gene expression, thereby aggravating disease severity and worsening clinical outcomes [1, 7–9]. Therefore, investigating the concomitant changes in the transcriptional regulation of circadian clock genes following stroke is crucial to identify novel intervention targets for stroke treatment and functional recovery.

The circadian rhythm is driven by core clock genes, with brain and muscle ARNT-Like protein 1 (*Bmal1*) being essential for maintaining the 24-hour cycle [10, 11]. Researchers have established a real-time monitoring system by transducing U2OS cells with lentiviral vectors carrying *Bmal1*-dluc or Period Circadian Protein 2(*Per2*)-dluc reporter genes, enabling the visualization of peripheral cellular rhythmicity [12–14]. Clinical evidence demonstrates that patients with sleep phase delay disorder exhibit significantly prolonged endogenous body temperature rhythm cycles [15], and individuals with distinct chronotypes show varying circadian rhythm periodicities [16]. Notably, while the suprachiasmatic nucleus (SCN) orchestrates global rhythmicity, peripheral cells possess autonomous circadian mechanisms. Serum from lung cancer patients prolongs the *Bmal1* oscillation period in U2OS cells [17], and serum from aged individuals alters fibroblast rhythmic properties [18], suggesting the presence of circulating factors that coordinate central-peripheral rhythm coupling. The synchronization and stability of central and peripheral rhythm systems following stroke warrant further investigation.

Emerging research focuses on brain functional connectivity(FC) changes in stroke and circadian rhythm-related disorders. Resting-state FC studies in delirium patients reveal enhanced SCN-dorsal anterior cingulate cortex connectivity but reduced connectivity with the posterior cingulate cortex, parahippocampal gyrus, cerebellum, and thalamus, indicating sleep/wake cycle disturbances may reorganize brain networks [19]. Stroke patients with sleep apnea exhibit thalamic sensory processing circuit hyperactivity, further linking rhythm abnormalities to neurological impairment [20]. Insomnia and sleep deprivation studies highlight dynamic interactions between circadian rhythm, sleep homeostasis, and brain FC [21, 22]. Night shift nurse studies demonstrate associations between enhanced hypothalamic-brain FC and circadian rhythm disruptions [23], while bipolar disorder research underscores the tight coupling between sleep/rhythm systems and brain function [24].

Despite these advances, it remains unknown whether circulating factors in stroke patients directly affect the molecular clock in peripheral cells, and whether such peripheral clock changes correlate with alterations in brain FC. To bridge this gap, the present study employed a human osteosarcoma U2OS cell line stably expressing *Bmal1* as a model to investigate the specific impact of stroke on peripheral rhythms by monitoring the oscillation cycles of cellular *Bmal1*following treatment with serum from ischemic stroke patients. Simultaneously, resting-state functional magnetic resonance imaging (rs-fMRI) based on seed-based analysis was utilized to explore the intrinsic relationship between alterations in the length of the *Bmal1* circadian period and brain FC. The primary objective was to test whether stroke serum alters peripheral circadian periodicity and whether such alteration is associated with stroke-related brain network changes.

## Methods

This study was conducted at the Neurorehabilitation Department of China Rehabilitation Research Center between January 2018 and July 2019. The Ethics Committee of the China Rehabilitation Research Center approved all research procedures, and it was conducted according to the Declaration of Helsinki (CRRC-IEC-RF-SC-005-01).

### Participants recruitment

Participants undergoing stroke rehabilitation were recruited from inpatient services at the Neurorehabilitation Department of the China Rehabilitation Research Center. Additionally, right-handed healthy controls matched for age and gender with stroke patients were recruited through community-based enrollment. The attending physician evaluated whether the study candidates met the enrolment criteria and explained the study procedures and goals to the participants and their families.

All participants provided written informed consent for enrollment in the study. The participants provided general information (age, occupation, cultural level, and medical history) and underwent clinical evaluation, physical examination, blood sample collection, and MRI.

### Enrollment

#### Inclusion criteria

Inclusion criteria for healthy participants were as follows: (1) age 18–60 years; (2) right-handedness; (3) good physical health without medical comorbidities; 4)ability to undergo MRI examinations;5)no travel across time zones or sleep disorders for at least 6 months before participation; 6) provision of written informed consent.

Inclusion criteria for stroke patients were as follows: (1) age 18 ~ 60 years; (2) right-handedness; (3) first episode of ischemic stroke; (4) the course of the stroke is greater than 2 weeks, and the condition is stable; (5) no history of sleep disorder before stroke; (6) no serious cognitive impairment and the score of Mini-Mental State Examination is 30; (7) cranial MRI confirmation of lesion located in the middle cerebral artery territory; and (8) provided written informed consent.

#### Exclusion criteria

For stroke patients:1) history of other neurological diseases; 2) patients with severe aphasia or dementia who could not cooperate with the examination; 3) use of hypnotic, anti-anxiety drugs or antidepressants before stroke; and 4) poor compliance and inability to complete sample collection and MRI evaluation.

Exclusion criteria for controls encompassed shift work, transmeridian travel within 6 months, anxiety/depression disorders, sleep disturbances, and structural abnormalities on cranial MRI.

### Demographics and stroke severity assessment

All participants completed a standardized comprehensive questionnaire capturing demographic information, including age, gender, education level, occupational status, and other relevant sociodemographic parameters. Sleep quality was assessed among all participants using the Chinese version of Pittsburgh Sleep Quality Index (PSQI) [25, 26].The severity of stroke was evaluated by National Institute of Health Stroke Scale (NIHSS) [27, 28].

### Sample collection

Fasting peripheral venous blood was collected daily at 6:00 (UTC + 8) within 24 h of enrollment. This fixed morning time point was chosen to minimize circadian phase variability between individuals, allowing phase-controlled comparisons of serum effects on Bmal1 period length. Blood samples were incubated at room temperature (22–25 °C) for 30 min; after centrifugation (5000 ×g for 10 min), the serum was collected and cryopreserved at − 80 °C until use.

### Synchronization and measurement of the circadian period

The bioluminescence recording and data analysis protocol has been described previously [12, 17, 29]. Lentiviral *Bmal1-Luc* reporters were introduced into U2OS cells via lentivirus-mediated infection. Clonal cell lines of homogenous cell populations were obtained by single-cell sorting and cloning in 96 well plates.

1. Cell Culture: U2OS cells were maintained in Dulbecco’s Modified Eagle Medium (DMEM) supplemented with 10% fetal bovine serum (FBS), 1% antibiotics, and 1% GlutaMAX (Thermo Fisher Scientific, Waltham, MA)until reaching full confluence.
2. Cell Synchronization: Confluent cells were transferred to high-serum synchronization medium containing 50% FBS, 1% antibiotics, 1% GlutaMAX, and 45 mM NaHCO3 for 2 h to synchronize circadian rhythms.
3. Conditioned Medium (CM) Preparation: Phenol red-free DMEM was supplemented with 1% antibiotics, 1% GlutaMAX, 1% luciferin, 4.5 mM NaHCO3, and 10 mM HEPES, adjusted to pH 7.2, to generate experimental CM.
4. Real-Time Reporter Assay Preparation [13, 17]: To investigate human serum effects on circadian periodicity, U2OS cells were transferred to CM containing 10% participant serum. Control assays using CM with 10% FBS were conducted in parallel to establish baseline circadian rhythms and validate experimental quality control.
5. Bioluminescence Measurement and Data Analysis: Continuous bioluminescence monitoring was performed over 7 days using LumiCycle equipment (Actimetrics, IL, USA). Bioluminescence data were analyzed through LumiCycle Analysis software to determine circadian period lengths [17].

**Fig. 1:**
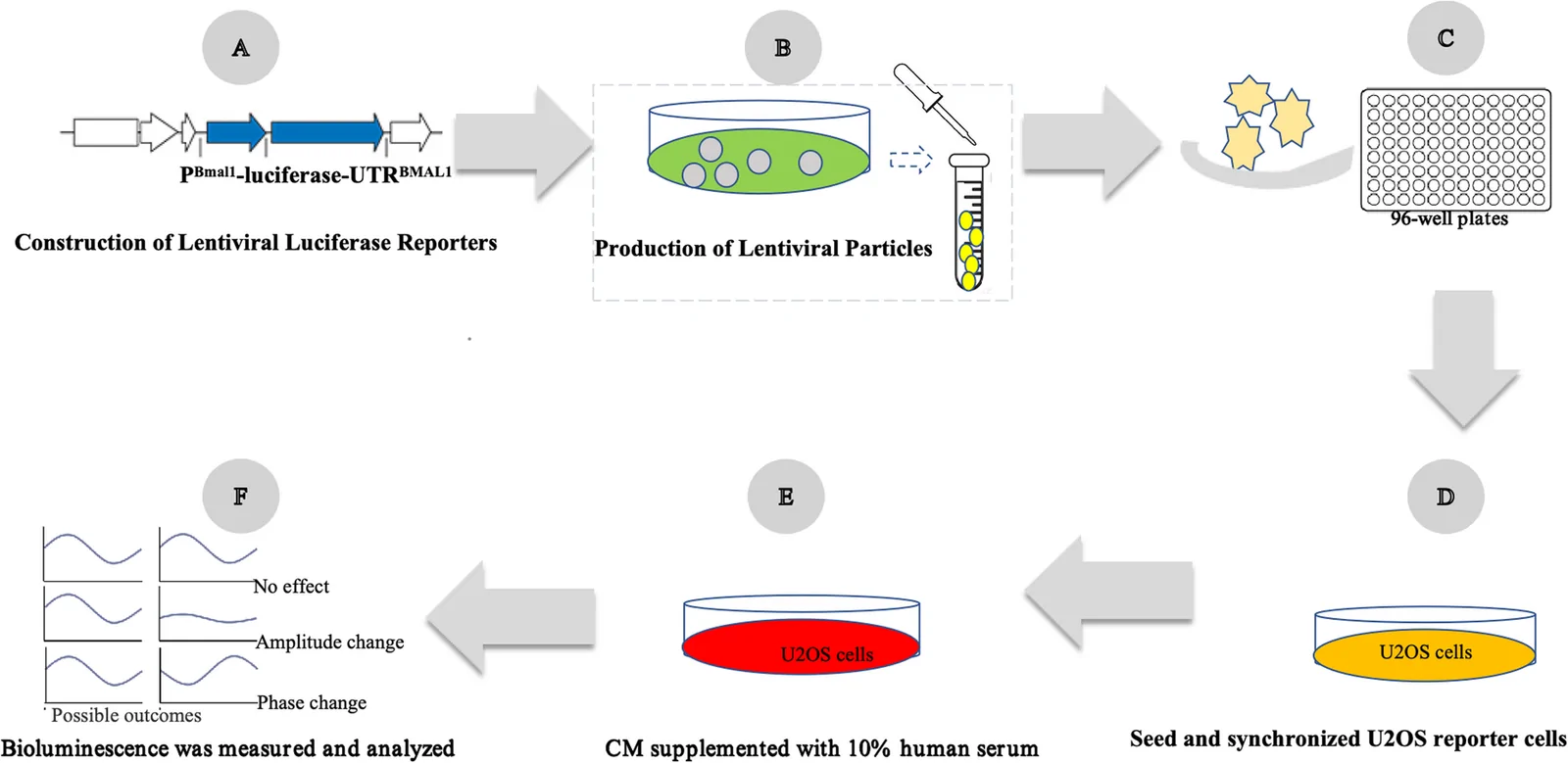
Schematic diagram of autonomous circadian clock rhythm luciferase bioluminescence reporters of human osteosarcoma (U2OS) cells. **A** Preparation of *Bmal1-Luc* plasmid; **B** Production of Lentiviral Particles; **C** Infection of U2OS Cells; **D** Seed and synchronized reporter cells; **E** Transfer of reporter cells into conditioned medium containing 10% human serum; **F** Bioluminescence measurement and data analysis using the LumiCycle system, showing possible outcomes (period lengthening, amplitude change, or phase change). Note: CM, conditioned medium

Raw data were initially fitted to baseline, and baseline-subtracted data were fitted to a sine wave from which the parameters were determined. A goodness-of-fit of > 80% was usually achieved for samples that showed persistent rhythms. The first data cycle was excluded from the analysis because of high transient bioluminescence upon medium change. All the steps are shown in Fig. 1.

### Magnetic resonance imaging (MRI) examination

All participants’ MRI scans were performed at 4 p.m. (UTC + 8) within 48 h of enrollment. Imaging data were acquired using a Philips Achieva 3-T MRI scanner (Philips Healthcare, Best, Netherlands) with an eight-channel head coil while the participants were conscious. The scanning protocol has been described in our previous studies [23]: 13 min 19 s; repetition time (TR) = 2,000 ms; echo time (TE) = 30 ms; flip angle = 90°; voxels = 3 × 3 × 3 mm^3^; number of slices = 50; gap = 0.6 mm, FOV = 220 mm × 220 mm; and matrix = 80 × 80. Standard high-resolution 3D anatomical T1-weighted scans (sagittal acquisition, TR = 8.2 ms, TE = 3.2 ms, flip angle = 8°, isotropic voxel = 1 mm.All imaging acquisitions were uniformly conducted by a single highly experienced radiologist.

### Neuroimaging acquisition and preprocessing

**Fig. 2:**
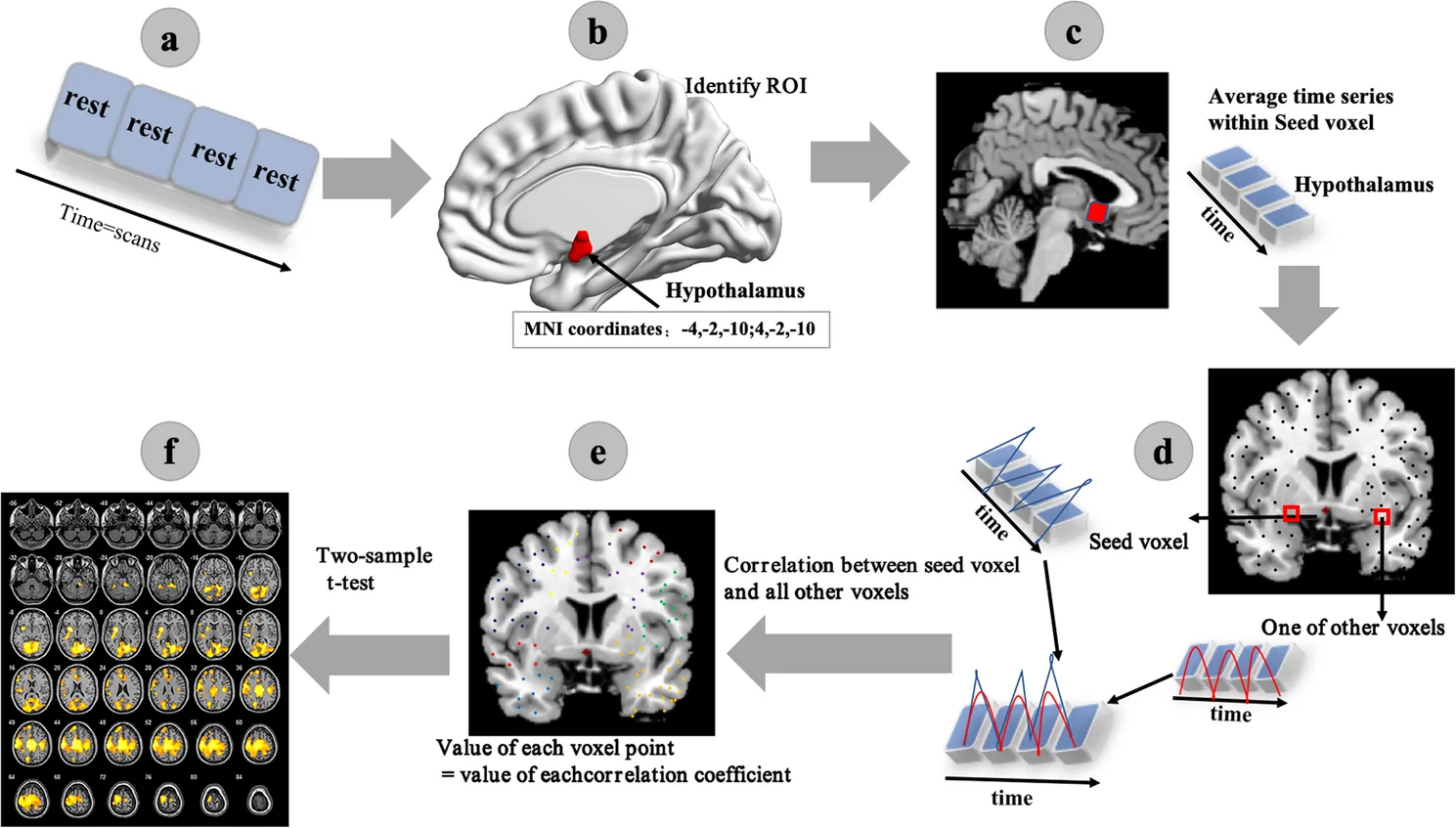
Neuroimaging processing and seed-based whole-brain functional connectivity analysis. **a** Neuroimaging acquisition and preprocessing; **b** The hypothalamus was used as region of interest (ROI). The MNI coordinates of the hypothalamus were (− 4, − 2, −10) and (4, − 2, −10); (c) Average time series within the seed region were calculated; **d**,** e** Voxel-wise Pearson’s correlation coefficients between the seed and the whole brain were computed to generate functional connectivity (FC) maps; **f** Voxel-wise group comparisons were performed using the general linear model with two-sample t-tests in SPM12, corrected by Gaussian Random Field (cluster-level *p* < 0.05)

MRI data underwent preprocessing and advanced analysis using DPABI (Data Processing & Analysis for Brain Imaging) [23, 30]. Imaging data in DICOM (Digital Imaging and Communications in Medicine) format were converted to NIFTI (Neuroimaging Informatics Technology Initiative) format. Using the echo-planar imaging (EPI) template from the Montreal Neurological Institute (MNI), image quality was systematically inspected for each dataset, followed by slice timing correction and head motion correction. After Gaussian smoothing and low-frequency filtering, regions of interest (ROIs) were defined based on the Anatomical Automatic Labeling (AAL) atlas [31]. Bilateral hypothalamic regions were selected as seed points for subsequent analyses. Whole-brain Pearson’s correlation coefficients between the seed and all other voxels were computed to generate FC maps. These maps were smoothed with an 8-mm full-width at half-maximum (FWHM) Gaussian kernel. Voxel-wise group comparisons between stroke patients and healthy controls were performed using the general linear model (GLM) as implemented in Statistical Parametric Mapping 12 (SPM) software, with two-sample t-tests. Multiple comparison correction was applied using Gaussian Random Field (GRF) theory, and significant differences were determined at a cluster-level threshold of *p* < 0.05 (as seen in Fig. 2).

### Statistical analysis

This exploratory, hypothesis-generating study aims to identify potential associations between peripheral clock alterations and brain FC in stroke patients; findings require replication in larger confirmatory studies. Statistical analyses were performed using SPSS version 26.0 (IBM, Armonk, NY, USA). Normality was assessed via Kolmogorov–Smirnov (K–S) tests coupled with histogram inspections. Normally distributed continuous variables are presented as mean ± standard deviation (SD), and group comparisons were conducted using independent-samples t-tests. Non-normally distributed data are presented as median and interquartile range (IQR) [M(P25, P75)] and were compared using Mann–Whitney U tests. All tests were two-tailed, with statistical significance defined as *p* < 0.05.

To control the type I error risk in demographic and clinical variable comparisons, the Benjamini–Hochberg correction was applied. For fMRI voxel-wise comparisons, GRF correction was applied at the cluster level (*p* < 0.05). For the subsequent Spearman correlation analyses between FC values and the *Bmal1* period, only two specific ROI pairs (hypothalamus-right calcarine, hypothalamus-right Rolandic operculum) were tested. These ROIs were selected a priori based on the group contrast maps from the fMRI analysis; therefore, no additional correction was applied for these two correlations. Nonetheless, given the exploratory nature of the study, all p-values should be interpreted cautiously. Spearman’s rank correlation analysis was employed to explore associations between the circadian oscillation period length of *Bmal1* in U2OS cells (independent variable) and post-stroke alterations in corresponding brain functional connectivity (dependent variable).

## Results

### Demographic characteristics of the study population

This study successfully enrolled 34 age- and sex-matched participants, comprising 17 healthy controls and 17 stroke patients. Statistical analysis demonstrated no significant intergroup differences in age, sex distribution, or education duration. Stroke patients exhibited a mean disease duration of 39.824 ± 21.381 days, with right-sided hemiplegia being the predominant presentation. Detailed demographic characteristics are provided in Table 1.

**Table 1:** Demographic and clinical characteristics of all subjects

Characteristic	Healthy controls (*n* = 17)	Stroke patients (*n* = 17)	t/χ2-score	*P*-value
Age (years)	51.88 ± 10.83	54.41 ± 10.42	0.694	0.493
Disease duration (days)	N.A	39.82 ± 21.38	-	-
Education (years)	16.06 ± 3.86	13.41 ± 4.86	1.757	0.089
Gender, male, n (%)	9(52.9)	11(64.7)	0.486	0.728
Lesion location, n (%)				
– Right basal ganglia region	N.A	3(17.6)	-	-
– Left basal ganglia region	N.A	10(58.8)	-	-
– Left basal ganglia involving cortex	N.A	4(23.5)	-	-
Hemiplegic side, n (%)				
– Right	N.A	14(82.4)	-	-
– Left	N.A	3(17.6)	-	-
NIHSS score	N.A	8.47 ± 3.08	-	-
PSQI score	2.35 ± 1.50	5.00 ± 1.37	5.379	< 0.001
*Bmal1* circadian period (hours)	23.81 ± 0.48	24.32 ± 0.41	3.352	0.002
Amplitude (a.u.)	2.02 ± 0.457	2.08 ± 0.249	0.490	0.628

Data are presented as mean ± SD or n (%)

*N.A*, not applicable, *NIHSS* National Institutes of Health Stroke Scale, *PSQI* Pittsburgh Sleep Quality Index, *a.u*. arbitrary unit

### Serum from patients affected by stroke lengthens the daily oscillation period of *Bmal1*

The length of the daily oscillation period of *Bmal1* in U2OS cells treated with media containing serum from healthy controls and stroke patients was 23.81 ± 0.48 h and 24.32 ± 0.41 h, respectively (Table 1). Serum from stroke patients significantly lengthened the period of *Bmal1* transcriptional oscillation compared to serum from controls (t = 3.352, *p* = 0.002, 95% confidence interval [CI]: 0.202 to 0.828). PSQI scores were correalted with the daily oscillation period of *Bmal1* (*r* = 0.437, *p* = 0.01). The oscillation amplitude did not differ significantly between the two groups (stroke: 2.08 ± 0.25, control: 2.02 ± 0.46, *p* = 0.628).

### Abnormalities in hypothalamic functional connectivity and their correlation with *Bmal1* oscillation period

**Fig. 3:**
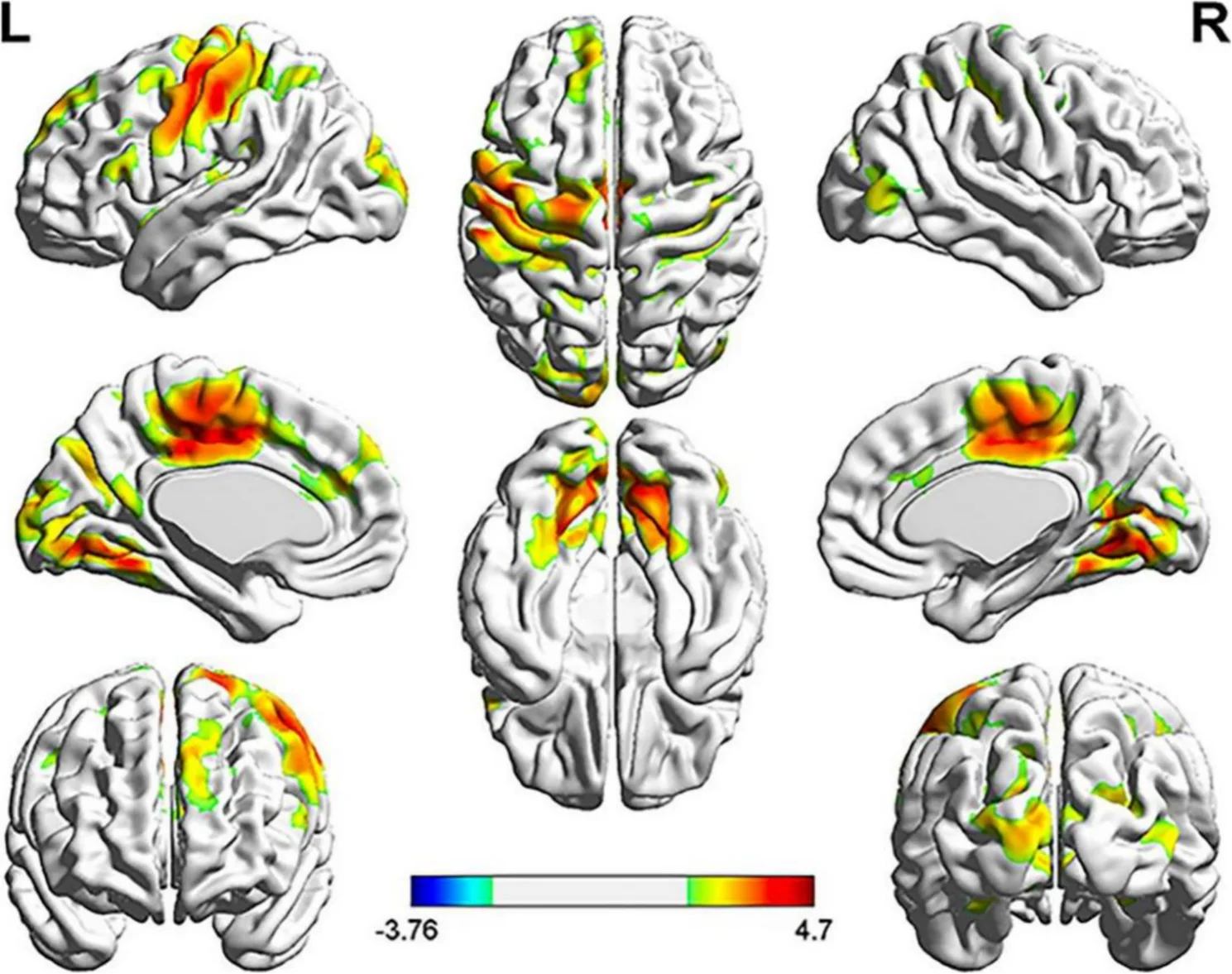
Brain regions showing significant differences in hypothalamic functional connectivity between stroke patients and healthy controls. Warm colors (red/yellow) indicate increased FC in stroke patients; cool colors (blue/green) indicate decreased FC in stroke patients. Results were thresholded at cluster-level *p* < 0.05 (GRF-corrected). L, left; R, right

**Fig. 4:**
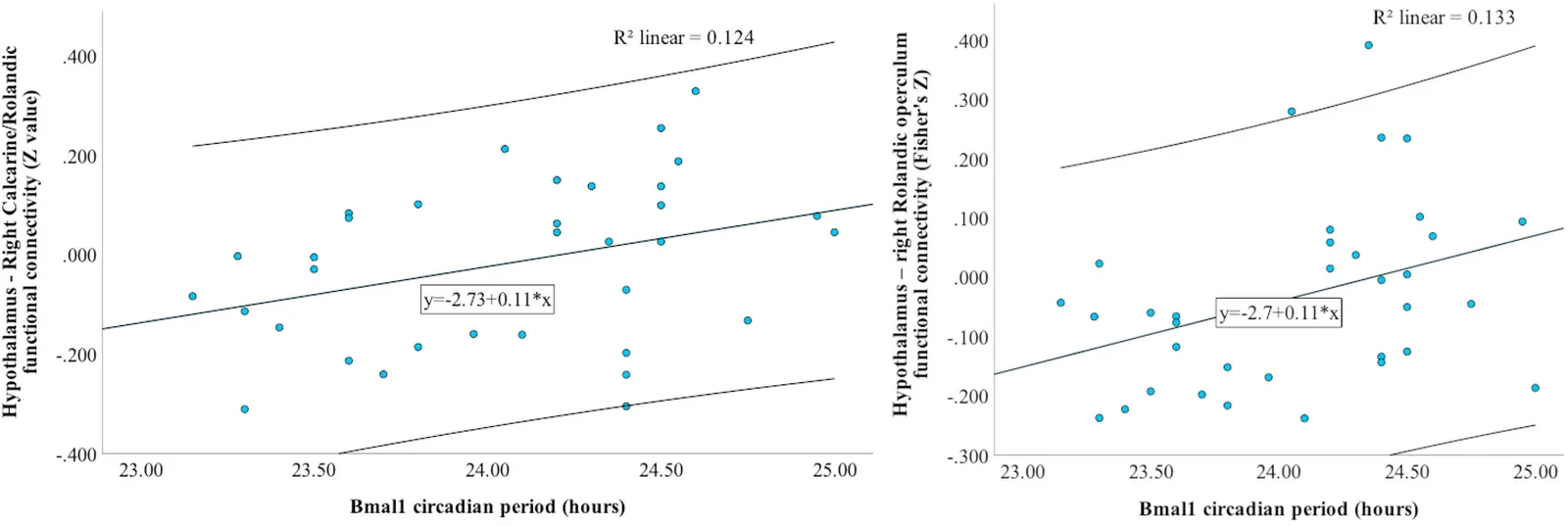
Correlation between Bmal1 oscillation period and hypothalamic functional connectivity in stroke patients (*n* = 17). **A** Scatter plot showing the positive correlation between *Bmal1* period and FC from the hypothalamus to the right calcarine cortex (ρ = 0.369, *p* = 0.032). **B** Scatter plot showing the positive correlation between *Bmal1* period and FC from the hypothalamus to the right Rolandic operculum (ρ = 0.393, *p* = 0.022). Solid lines represent linear regression fits, and shaded areas indicate 95% confidence intervals. FC values are Fisher’s Z-transformed

Compared to healthy controls, stroke patients exhibited significant brain FC alterations across multiple brain regions, encompassing the frontal lobe, bilateral occipital lobes, left basal ganglia, bilateral cingulate gyrus, and right Rolandic operculum (Fig. 3) After applying Benjamini-Hochberg correction for multiple comparisons, the bilateral occipital lobes emerged as the primary regions of divergence, particularly the lingual gyrus and calcarine cortex (*p* ≤ 0.025) (Table 2). Subsequent Spearman’s rank correlation analysis between FC changes and *Bmal1* oscillation period demonstrated that enhanced FC from the hypothalamus to the right calcarine gyrus (*r* = 0.369, *p* = 0.032) (Fig. 4A) and to the right Rolandic operculum (*r* = 0.393, *p* = 0.022) significantly correlated with prolonged *Bmal1* oscillation period (Fig. 4B).

**Table 2:** Brain regions with significantly different hypothalamic functional connectivity between stroke patients and healthy controls

Brain region (AAL)	Healthy controls (mean ± SD)	Stroke patients (mean ± SD)	t-value	*p*-value*	95% CI
Right calcarine	-0.099 ± 0.155	0.056 ± 0.146	2.996	0.005	(0.049, 0.259)
Left calcarine	-0.098 ± 0.107	0.060 ± 0.146	3.592	0.001	(0.068, 0.247)
Right lingual	-0.075 ± 0.166	0.071 ± 0.165	2.587	0.014	(0.031, 0.262)
Right Rolandic operculum	-0.089 ± 0.130	0.021 ± 0.175	2.068	0.047	(0.002, 0.217)

Data are presented as mean ± SD.  Bilateral calcarine and right lingual regions remained significant after Benjamini-Hochberg correction (*p* ≤ 0.025). The right Rolandic operculum was included based on a priori hypothesis from group contrast maps

*AAL* Anatomical Automatic Labeling, *CI* confidence interval

^*^p-values are uncorrected

## Discussion

In this study, we compared the peripheral *Bmal1* transcriptional oscillation period in U2OS cells treated with serum from stroke patients versus healthy controls. Our results showed that serum from stroke patients significantly lengthened the *Bmal1* oscillation period (24.32 ± 0.41 h vs. 23.81 ± 0.48 h, *p* = 0.002). Using resting-state fMRI, we found that stroke patients exhibited enhanced FC from the hypothalamus to the right calcarine cortex and right Rolandic operculum compared with healthy controls. Moreover, the degree of this connectivity enhancement correlated positively with the prolonged *Bmal1* period (*r* = 0.369, *p* = 0.032; *r* = 0.393, *p* = 0.022). These empirical observations suggest an association between peripheral clock properties and stroke-related brain FC changes.

Previous studies have identified associations between core circadian gene polymorphisms and ischemic stroke risk. For example, the period circadian regulator 1 (Per1) rs2253820 variant increases stroke susceptibility [32]. Post-stroke abnormalities in peripheral circadian biomarkers, such as melatonin, cortisol, body temperature, and blood pressure, are frequently reported [33–35]. Upregulation of cryptochrome 1 (Cry1) expression may reflect circadian dysregulation in stroke patients [36], while particulate air pollution modulates stroke outcomes partly via Cry1/Cry2 methylation status [37]. Furthermore, experimental evidence indicates that circadian phase modulates neuronal injury and neuroprotective efficacy in stroke models [38]. The observed circadian phenotype—prolonged period without a statistically significant change in amplitude—suggests that circulating factors in post-stroke serum may preferentially affectcellular pathways that regulate period length. This possibility could help refine the search for underlying molecular mechanisms and is consistent with previous reports showing that serum from pathological conditions primarily affects circadian period [15, 17, 18].

Altered brain FC associated with circadian disruption has been described in populations without structural brain lesions, including delirium patients [19], sleep-deprived individuals [39], and night-shift nurses [23]. In stroke patients, however, brain structural damage adds complexity. Lesions in the secondary visual cortex, frontotemporal parietal lobe, occipital lobe, and thalamus have been linked to sleep-wake disturbances [20], and post-stroke insomnia is associated with altered regional brain activity [40]. Sacchetti et al. reported enhanced thalamic functional connectivity in post-stroke sleep apnea [20]. Our study extends these findings by demonstrating that hypothalamic connectivity changes in stroke patients correlate with a molecular clock readout (*Bmal1* period length), providing a potential link between peripheral circadian signals and central network reorganization.

The present study adopted rigorous methodological controls, matching participants across sex, age, education, and MRI acquisition times to isolate stroke-specific effects. This matching is particularly important given that both sex and circadian phenotypes have been shown to influence brain functional organization [41, 42]. Based on our correlational data, we hypothesize that circulating bioactive factors in stroke serum—possibly inflammatory cytokines or metabolic byproducts—may lengthen the Bmal1 oscillation period and simultaneously affect hypothalamic function, leading to altered brain FC [43, 44]. However, we did not measure any inflammatory markers (e.g., C-reactive protein, interleukin-6, white blood cell count) or other candidate factors. Therefore, this hypothesis remains untested, and alternative explanations (e.g., lesion-induced network reorganization independent of circadian mechanisms) cannot be excluded. Direct causal evidence is lacking, and our findings should be considered hypothesis-generating.

Several important limitations warrant explicit acknowledgment. First, the small sample size substantially limits statistical power, particularly for neuroimaging correlation analyses. Many of the reported correlations may be unstable and susceptible to type I and type II errors. Thus, these exploratory results require replication in larger independent cohorts. Second, although all stroke patients had lesions within the middle cerebral artery territory, we did not quantitatively control for lesion volume or exact anatomical location. Structural damage can directly influence FC patterns, and the observed hypothalamic FC changes may partly reflect lesion-induced reorganization rather than circadian-related effects. Future studies with voxel-based lesion-symptom mapping are needed. Third, blood was collected only once at 6:00 AM. While this fixed time point minimized inter-individual phase variability and allowed phase-controlled comparisons of Bmal1 period length, it does not capture circadian amplitude, acrophase, or phase shifts. Therefore, our study does not constitute a full circadian rhythm assessment. Multi-timepoint sampling (e.g., every 4–6 h over 24–36 h) is required to comprehensively characterize post-stroke circadian alterations. Fourth, we applied GRF correction for voxel-wise fMRI comparisons [45] and Benjamini-Hochberg correction for region-level analyses [46]. The two correlation analyses were limited to a priori ROIs based on group contrast maps; thus no additional correction was applied. Nevertheless, given the exploratory nature of the study, all p-values should be interpreted cautiously. Fifth, we did not collect or analyze any laboratory markers of inflammation. Given the well-established role of post-stroke inflammation in circadian disruption [43, 44], the lack of such data prevents us from determining whether inflammatory responses underlie the observed peripheral clock changes. This omission is a key limitation.

Future directions. Beyond inflammatory markers, complementary neurophysiological tools such as electroencephalography (EEG) could provide valuable insights into post-stroke circadian dynamics. EEG offers high temporal resolution and can capture sleep architecture, brain state fluctuations, and neural oscillatory patterns that reflect circadian influences on cortical excitability and recovery [47, 48]. Integrating resting-state and sleep EEG recordings in future longitudinal studies may help to better characterize the neurophysiological signatures of circadian disruption after stroke and could serve as an additional bridge between peripheral circadian signals and central network reorganization.

## Conclusions

In this exploratory, hypothesis-generating study, we found that serum from stroke patients lengthens the *Bmal1* circadian period in U2OS cells and that this lengthening correlates with enhanced hypothalamic FC to the right calcarine and Rolandic operculum. These findings provide preliminary evidence of a possible link between peripheral clock properties and central network reorganization after stroke, but causality remains unproven. Owing to the small sample size, lack of inflammatory marker measurement, and single-time-point sampling, our results are preliminary and require replication in larger independent cohorts. Future work should integrate multi-timepoint biomarker sampling, lesion-symptom mapping, and inflammatory profiling to further investigate the mechanisms underlying post-stroke circadian and brain functional changes.

## Data Availability

The datasets generated and/or analysed during the current study are not publicly available due to ethical restrictions (protecting human participant privacy). They can be requested from the corresponding author on reasonable request.

## Abbreviations

AAL: Anatomical automatic labeling
Bmal: Brain and muscle arnt-like
CM: Culture medium
DMEM: Dulbecco’s modified Eagle’s medium
CI: Confidence interval
Cry1: Cryptochrome 1)
DICOM: Digital Imaging and Communications in Medicine
DPABI: Data Processing & Analysis for (Resting-StateBrain Imaging)
EPI: Epipolar-plane image
FBS: Fetal bovine serum
FC: Functional connectivity
fMRI: Functional magnetic resonance imaging)
FWHM: Ffull width at half maximum
MN: Motor network
MNI: Montreal Neurological Institute
NIHSS: National Institute of Health Stroke Scale
Per: Period gene family
PSQI: Pittsburgh sleep quality index
ROI: Region of Interest
SCN: Suprachiasmatic nucleus
UTC: Coordinated Universal Time
